# Surface Properties of Medium-Entropy Alloy Coatings Prepared through a Combined Process of Laser Cladding and Ultrasonic Burnishing

**DOI:** 10.3390/ma15165576

**Published:** 2022-08-13

**Authors:** Xuehui Shen, Chang Liu, Baolin Wang, Yu Zhang, Guosheng Su, Anhai Li

**Affiliations:** 1 School of Mechanical Engineering, Qilu University of Technology (Shandong Academy of Sciences), Jinan 250353, China; 2 Shandong Institute of Mechanical Design and Research, Jinan 250031, China; 3 Key Laboratory of High Efficiency and Clean Mechanical Manufacture of MOE, School of Mechanical Engineering, Shandong University, Jinan 250061, China

**Keywords:** medium-entropy alloy, laser cladding, ultrasonic burnishing, surface roughness, wear resistance

## Abstract

The preparation of functional coatings on metal substrates is an effective method to enhance the surface of steel structures with good serviceability in applications for engineering parts. The objective of this research is to analyze the surface properties of two sorts of medium-entropy alloy (MEA) coatings prepared by laser cladding. After cladding, the two prepared coatings were strengthened by ultrasonic burnishing (UB) treatment. Cladding coating samples before and after being UB-treated were comparatively tested in order to investigate the process effects of UB. When compared with corresponding untreated coating samples, the roughness values of the two sorts of UB-treated samples were decreased by 88.7% and 87.6%, the porosities were decreased by 63.8% and 73.4%, and the micro-hardness values were increased by 41.7% and 32.7%, respectively. Furthermore, the two sorts of UB-treated coating samples exhibited better mechanical properties and wear resistance than corresponding untreated samples.

## 1. Introduction

In recent years, superior to most traditional alloys [1], multi-principle-element alloys (MPEAs) show excellent properties, such as high strength [2], high hardness [3], high thermal stability [4], and excellent corrosion resistance [5]. Hence, they have a great potential to be applied in the fields of energy, aerospace, pipeline engineering, and so on.

A high-entropy alloy is composed of five or over five kinds of elements with equal or nearly equal molar ratios, and the content of each element is between 5 and 35 at.%. As is well known, high-entropy alloys have four main effects, i.e., the lattice distortion effect, cocktail effect, high-entropy effect, and hysteresis diffusion effect [6,7], and thus show significant performance in many aspects. MEAs are derived from high-entropy alloys, composed of three or four elements in accordance with equal or nearly equal molar ratios. Until now, three kinds of single-phase high-/medium-entropy alloys with face-centered cubic (FCC) structures, namely, CoCrNi, FeCoNiCr, and NiCoCrFeMn, have been widely studied [8,9]. Among these popular high-/medium-entropy alloys with FCC structures, CoCrNi has the optimal strength and toughness [10], as well as unique low-temperature fracture toughness surpassing other known materials [11]. As a result, it has become one of the new materials attracting the most attention.

Generally, the preparation of MEA is mainly based on casting and sintering. Moreover, the main elements in MPEAs are usually expensive. These two aspects hinder the practical application of MEAs. Laser cladding is an advanced surface coating preparation technique that has been widely used to modify the surface properties of engineering parts [12,13]. During laser cladding, alloy powders and the metal matrix surface simultaneously melt using a high power density laser beam, and then the liquid metal rapidly solidifies to form a cladding layer metallurgically bonded with the matrix. MEAs can be coated on surfaces of low-cost material substrates, with a surface performance reaching or exceeding that of the whole medium-/high-entropy alloy, which is an effective method to promote the further application of alloys with multiple principal elements.

Recently, some scholars have carried out research on the preparation and property investigation of high-entropy alloy laser cladding coating. Zhang et al. [14] prepared a TiZrNbWMo refractory high-entropy alloy coating by laser cladding on the surface of a 45 steel substrate, and then annealed the sample for 20 h at 800~1200 °C. The results showed that the microstructures of the cladding coating were mainly BCC phase and a small amount of *β*-TixW1-x precipitated phase. After being annealed, the microstructure of the BCC phase basically remained stable, illustrating that the prepared cladding coating had higher thermal stability. Simultaneously, the proportion of precipitated phase increased, which suggested that the micro-hardness was improved. Liang et al. [15] prepared AlCrFeNi2W0.2Nbx (x = 0.5, 1.0, 1.5, 2.0) cladding coatings on the surfaces of SS304 substrates, and conducted a wear test. The results showed that the principal phase of the cladding coating was the single BCC solid-solution phase. As x was equal to 1.0, 1.5, and 2.0, there appeared a Laves phase, and the micro-hardness of the cladding coating increased with the Nb content increasing.

However, there exists a “rapid heating and sudden solidification” characteristic in the laser cladding process. The substrate material is usually different from that of cladding coating; accordingly, their thermal expansion coefficients are greatly different, resulting in the generation of structural stress and thermal stress. Moreover, the internal pores and defects could cause the uneven distribution of the hardness of the cladding coating, thus affecting the service performance of the cladded components. UB is a kind of surface-strengthening technique to generate a gradient nanostructure surface layer and fine grains [16]. In addition to the significant decrease in surface roughness, UB treatment can induce compressive residual stress in near-surface materials. Thus, by UB treatment, a gradient structure with more refined grains of treated material could be obtained, achieving an improvement in the surface integrity and mechanical properties of the materials [17,18,19]. At present, UB technology has been widely applied to many materials, such as iron-base alloy, Ti, commercial pure aluminum, Cu, and Ni alloys [20,21]. Previous reports have demonstrated that ultrasonic surface burnishing treatment can produce a nearly polished surface, gradient nanostructured surface layer, and high residual compressive stress on the friction stir-welded joint of a 7075-T651 aluminum alloy. Furthermore, the fatigue property of the joint after treatment was enhanced and the fatigue life was extended by two orders of magnitude [22]. Applying UB technology on a Cu-10Ni alloy, a gradient nanostructured surface layer was obtained with an 80% increase in surface hardness. Additionally, the Cu-10Ni alloy has better corrosion resistance in 3.5wt% solution due to the formation of surface passivation film promoted by the nanoparticle surface during the corrosion process [23]. However, there are few studies on the UB strengthening of MEA laser cladding coating.

Herein, the aim of our study is to improve the wear resistance of a GCr15-bearing steel surface. A laser cladding technique was applied to prepare two kinds of MEA cladding coatings, i.e., CoCrNi coating and FeCoNiCr coating. After cladding, UB strengthening treatment was conducted on the prepared laser cladding coatings. By comparison, the influences of UB treatment on the surface integrity, mechanical, and tribological properties of the cladding coating were studied, and thus some conclusions were drawn.

## 2. Materials and Methods

### 2.1. Experiment Materials

**Figure 1:**
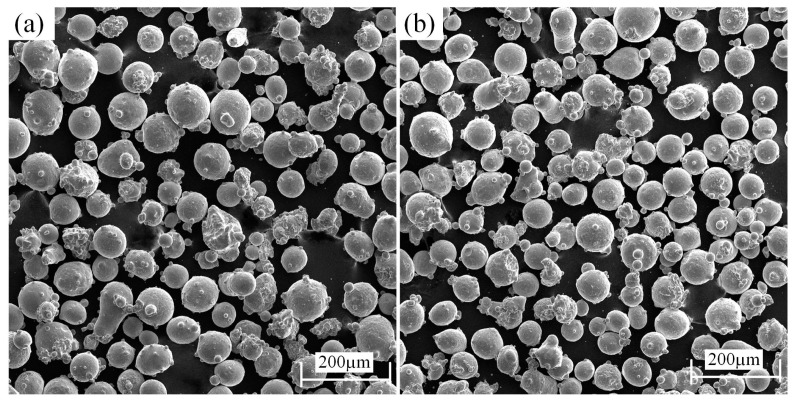
SEM images of MEA powder morphology, (**a**) FeCoNiCr, (**b**) CoCrNi.

The powders of FeCoNiCr and CoCrNi, purchased from Jiangsu VILORY New Material Technology Co., Ltd. (Xuzhou, China) were adopted with a purity of 99.9% and a particle size of 45~105 μm. The GCr15-bearing steel was used as the substrate with a size of Φ 55 mm × 60 mm. The chemical compositions of the FeCoNiCr and CoCrNi powders and substrate are listed in Table 1, respectively. Further, the approximately spherical microstructure of the powders is shown in Figure 1.

### 2.2. Sample Preparation

**Figure 2:**
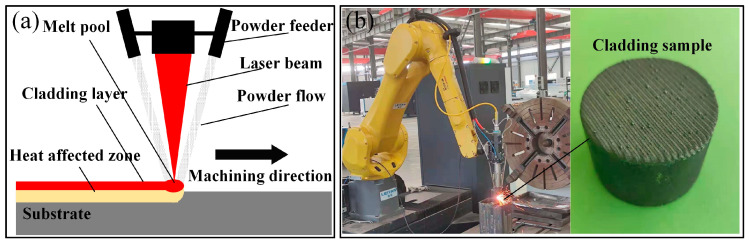
(**a**) Processing of laser cladding, (**b**) photos of laser cladding.

In order to prepare the samples, firstly, the GCr15 bearing steel substrate was ground with 180#, 400#, and 600# silicon carbide abrasive papers, respectively, to obtain a smooth surface with the oxide removal. Then, acetone was used to wipe the surface to remove contaminants such as oil stains. Finally, the LYRF-4000W robot laser cladding workstation and LYRF150 high-end cladding integration system were used to prepare the coatings on the substrate. The optimal laser cladding parameters are listed in Table 2, and the processing and photos of laser cladding are illustrated in Figure 2.

**Figure 3:**
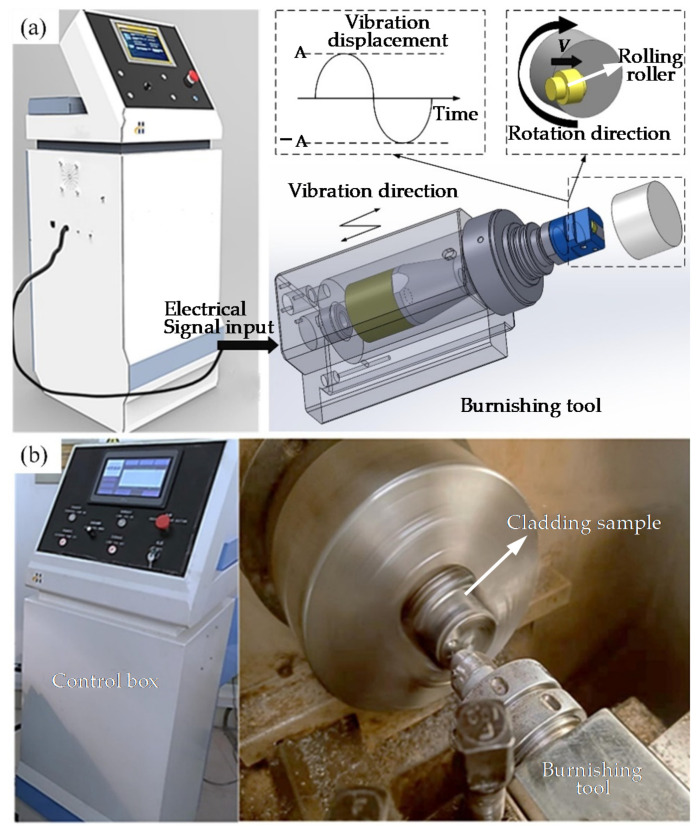
(**a**) Illustration of UB processing, (**b**) photos of the UB experiment setup.

After the laser cladding, the samples were firstly fixed on the CNC lathe for finish turning to obtain a smooth surface. Then, the UB process was conducted on the sample at room temperature. During the experiment process, the static pressure can be obtained by adjusting the extrusion depth of the lathe. The specific parameters are listed in Table 3. The UB processing and experimental setup are presented in Figure 3a,b, respectively.

A total of five different samples were prepared for the experiment, as follows. The substrate sample was briefly described as HT(GCr15) as a control sample. Two coating samples, prepared by first laser cladding and then turning finely, were briefly described as LC(FeCoNiCr) and LC(CoCrNi). Furthermore, another two coating samples, prepared by laser cladding, turning finely, and UB treatment, in sequence, were briefly described as UB(FeCoNiCr) and UB(CoCrNi). All samples were cleaned in an ultrasonic cleaner for 10 min after being treated.

### 2.3. Surface Morphology and Microstructure Characterization

The three-dimensional surface morphologies and roughness of different samples were tested using a white light interferometer with a surface 3D texture topography acquisition system (Contour Elite K, Bruker Company, Karlsruhe, Germany). In order to suppress measurement noise, various spline filters were applied to the data analysis of the surface topography [24,25,26]. Considering the level of the accuracy of measurement, the averaging method was used. Five different areas of each sample were selected for measuring the surface roughness and taking an average.

The surface microscopic morphologies of the laser cladding coatings of FeCoNiCr and CoCrNi MEAs were observed with an ultra-depth three-dimensional observation microscopic system (VHX-5000, KEYENCE CORPORATION, Osaka, Japan), as well as the microstructures after turning and UB treatment, subsequently, with the analysis for the observation results. The energy dispersive spectrometer (EDS, Phenom ProX, Phenom-World, Eindhoven, The Netherlands) was used to test the chemical composition of the samples. Before the observation, the samples via laser coating and UB treatment were ground with silicon carbide abrasive papers of 180#, 400#, 600#, 1000#, 1500#, and 2000#, respectively. Then, the sample surface was polished to a mirror finish without any scratch with a diamond polishing paste of W0.5. The hydrofluoric acid solution (hydrofluoric acid: nitric acid: water = 2:1:5) was used to etch the polished samples. The porosities of the coatings with different treatments were calculated using Image J software.

### 2.4. Micro-Hardness and Nano-Indentation Test

A micro Vickers hardness tester (HXD-1000TMC) was used to measure the hardness of the samples with various treatments at a load of 1.961 N (HV_0.2_) for 15 s. The interval between adjacent test points is 100 μm, and each test point was measured three times, taking the average to ensure the data accuracy.

The micro-/nano-mechanical properties of the sample surface were measured and characterized by a nano-indentation tester NHT (MTS, Bruker Company, Karlsruhe Germany) mechanical testing system for microscopic materials. The load of the nano-indentation test was 30 mN, with a loading/unloading speed of 15 mN/min and a load-holding time of 15 s. Moreover, the indentation test was repeated five times for each area to ensure the accuracy and reliability of the data.

### 2.5. Friction and Wear Test

Friction and wear performance tests were conducted on the substrate sample, as well as those coating samples after turning and UB treatment by an RTEC multifunctional friction and wear tester at room temperature, with a friction way of linear reciprocating motion. Before the test, the sample surface was slightly polished to make the surface smooth without an oxide layer. The counter pair was the GCr15 steel ball with a 7 mm diameter and 60 HRC. The load was 10 N, and the one-way friction distance was 4.5 mm. Furthermore, the reciprocating frequency was 2 Hz, and the friction and wear experiments hold for 15 min. The wear morphology and chemical composition were analyzed and tested by SEM and EDS. Meanwhile, a 3D Super Depth Digital Microscope (VHX-5000 KEYENCE CORPORATION, Osaka, Japan) was used to measure the wear profiles and calculate the wear loss in all testing cases.

## 3. Results and Discussion

### 3.1. Surface Roughness

**Figure 4:**
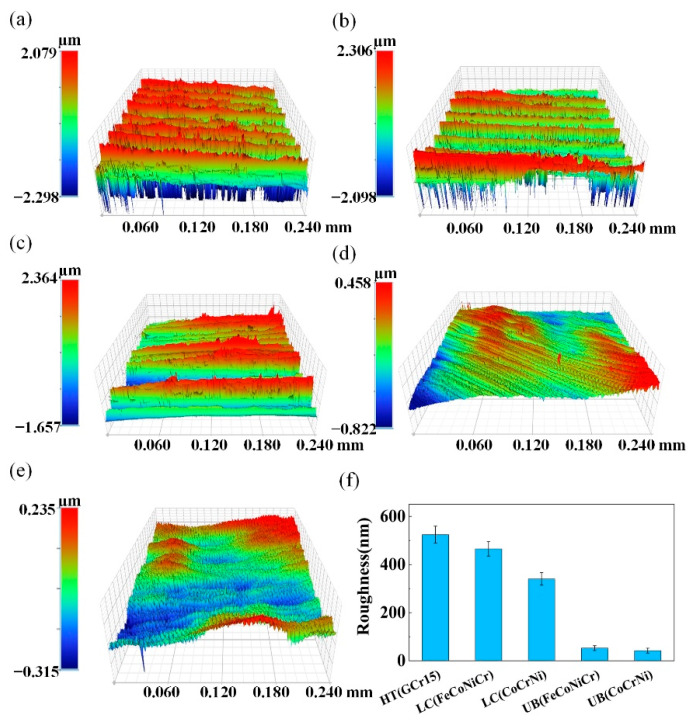
Three-dimensional surface roughness measurements of five samples. (**a**) HT(GCr15), (**b**) LC(FeCoNiCr), (**c**) LC(CoCrNi), (**d**) UB(FeCoNiCr), and (**e**) UB(CoCrNi). (**f**) Comparison of surface roughness parameter *S*a of five samples.

Figure 4 shows the comparison of the surface morphology and surface roughness for the substrate, untreated MEA coating, and UB-treated MEA coating sample surfaces. As can be clearly seen from Figure 4a–c, the turning contour of the substrate and untreated MEA coating samples were relatively stable, but there appeared obvious oscillation corrugations in the turning contour of the laser cladding samples compared with the UB-treated coating sample surfaces, wherein most areas were fattened, as shown in Figure 4d,e. The reason was that during the laser cladding process, the excessively fast cooling velocity resulted in the failure of gas escape in time, uneven residual stress distribution, and defects of void, inclusion, etc., thus affecting the quality of the turning surface. From Figure 4f, there appeared obvious oscillation corrugations on the surface of the laser cladding samples, but the surface roughness parameter *S*a (arithmetical average height of surface topography located reference surface [27,28,29]) of the laser cladding samples slightly decreased. Furthermore, the surface roughness parameter *S*a of the UB(FeCoNiCr) and UB(CoCrNi) samples were 52.612 nm and 42.098 nm, respectively, decreasing by 88.7% and 87.6%, compared with the *S*a = 465 nm of LC(FeCoNiCr) and the *S*a = 340 nm of LC(CoCrNi) samples, which were turned finely only. The reduction of *S*a would be beneficial to the improvement of surface properties. The surface roughness of samples via UB treatment became smaller, due to the following two aspects. On the one hand, the rolling head of UB caused the plastic deformation of the sample surface, making the material flow from surface peaks to troughs, thus eliminating turning traces. On the other hand, the rolling head intermittently contacts the sample surface, so the lubrication can fully flow to the working area. This can not only improve the strengthening effect but also reduce the adhesion between the roller and sample surface material. Hence, the surface roughness of the sample by UB treatment significantly decreases [30,31].

### 3.2. Porosity

With the rapid heating and cooling characteristics, dense and fine grains would be generated in the laser cladding coating. However, due to the excessively rapid cooling speed, the pores inside the laser cladding layer are always present in the laser cladding. To reduce the pores of various cladding coatings, many efforts have been made, especially in improving the laser process parameters [32].

The porosity not only affects the surface roughness but also reduces the mechanical properties of the coating surface, easily leading to high wear rates [33]. Therefore, it is an essential factor to evaluate the surface quality of laser cladding coatings.

**Figure 5:**
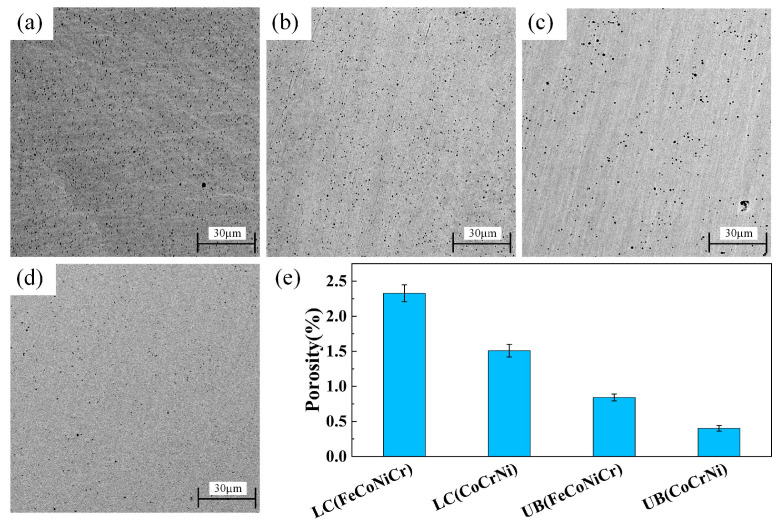
SEM images of coating sample surface morphologies. (**a**) LC(FeCoNiCr), (**b**) LC(CoCrNi), (**c**) UB(FeCoNiCr), (**d**) UB(CoCrNi), and (**e**) the porosities of coating samples.

Figure 5a–d shows the SEM images of the surface morphology of the four coating samples. As shown in Figure 5a–d, the numbers of pores in Figure 5c,d are significantly less than those in Figure 5a,b. Figure 5e shows the calculated porosities of the four coating samples. As exemplified in Figure 5e, compared with LC(FeCoNiCr) and LC(CoCrNi) coating samples, the surface porosities of the UB-treated coating samples decreased by 63.8% and 73.4%, respectively. Obviously, the UB treatment had a significant effect on decreasing the porosity of the laser cladding coatings. This was because, during the UB process, the external force caused the plastic deformation of the coating sample surface and then the plastic flow in the local deformation area. Accordingly, the pores and defects on the surface were filled, so that the porosity decreased, and then a smooth surface was formed.

### 3.3. Microstructure Characterization

**Figure 6:**
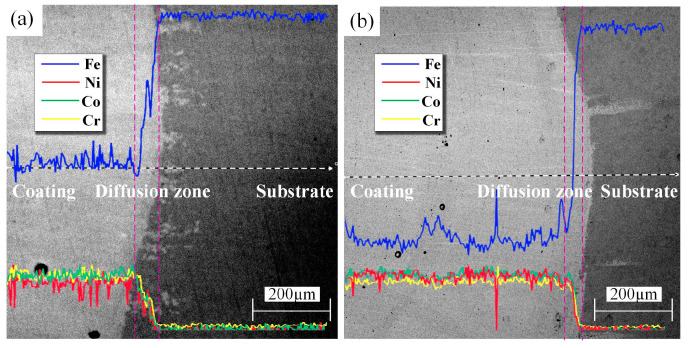
Elements distribution of samples. (**a**) FeCoNiCr, (**b**) CoCrNi.

Laser cladding can enable the metallurgical bonding between alloy powders and the metal substrate surface. Owing to the high temperature and element concentration difference, the element diffusion between the laser cladding coating and substrate could change the coating composition and microstructure, thus significantly affecting the coating surface properties. The EDS was used to characterize the chemical composition distribution of MEAs FeCoNiCr and CoCrNi coatings prepared by laser cladding. It was found from the characterization results in Figure 6 that the elements of Fe, Co, Ni, and Cr were evenly distributed in the coating, and there was an obvious transition with the substrate, illustrating the occurrence of metallurgical bonding between the coating and the substrate. Preparing the coating, the substrate had a relatively large dilution effect on the MEA due to the stirring of the molten pool. In the affected layer, the Fe content significantly increased, and near the surface, the element content was basically stable, but the Fe content still increased to a certain extent due to the dilution [34].

During the laser cladding process, the difference in the coefficients of thermal expansion between the substrate and laser cladding coating led to thermal stress generation. This caused a greater stress gradient from the substrate material to the cladding layer surface and an uneven distribution of internal stress in the cladding coating. Moreover, the internal pores and defects resulted in the uneven hardness of the cladding coating surface.

**Figure 7:**
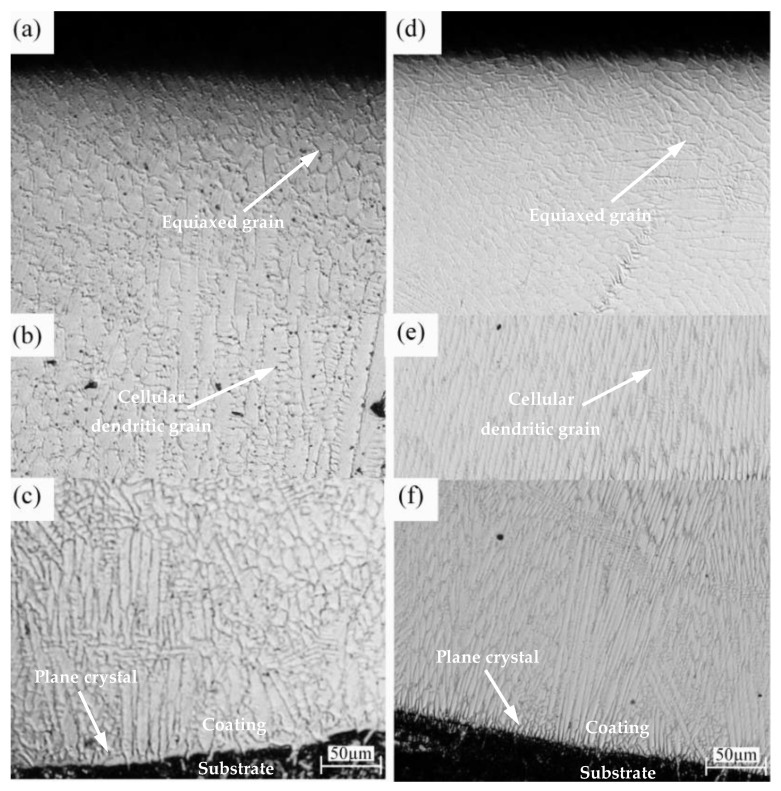
The metallographic images of the cross-sections of coating samples: (**a**–**c**) for LC(FeCoNiCr); (**d**–**f**) for LC(CoCrNi).

Figure 7a–c presents the metallographic structure images of the LC(FeCoNiCr) sample from the top of the coating to the substrate material, respectively. At the juncture of the coating with the substrate, the temperature gradient was the largest, and the grain growth rate was slow, leading to the formation of a plane crystal, as shown in Figure 7c. The cellular dendritic crystal zone was above the plane crystal, and there was a layer of coarse grains of cellular dendritic crystal, with an upward growth perpendicular to the substrate. When the plane crystal formed, the increase in substrate material temperature led to the decrease in the degree of subcooling, and thus the nucleation rate decreased but had little influence on the growth rate of the grains. The preferential growth direction of the grains is consistent with the opposite direction of the fastest heat dissipation direction; that is, upward diffusion from the substrate, which makes the grains grow upwards, perpendicular to the substrate, as shown in Figure 7b. The top grain was cooled in air with a greater degree of undercooling. The growth of columnar crystals was hindered at the solidification front. Moreover, as the degree of supercooling increased to a certain degree, many more new crystal nuclei appeared. They began to grow in all directions, formed an equiaxed-grain structure, and then prevented the growth of a columnar crystal. In addition, the heat flux at the top was greatly affected by the movement of the laser beam, leading to the growth direction of the equiaxed-grain structure parallel to the processing direction, as shown in Figure 7a.

Figure 7d–f shows the metallographic structure images of the LC(CoCrNi) sample from the top of the coating to the substrate material, respectively. Obviously, the top and bottom of the grain structures of CoCrNi MEA were similar to those of FeCoNiCr MEA. However, during the formation of the plane crystal at the bottom, the temperature gradient in the liquid phase at the interface front of CoCrNi MEA was smaller than that of FeCoNiCr MEA, resulting in a columnar crystal in the middle area rather than a cellular dendritic crystal.

**Figure 8:**
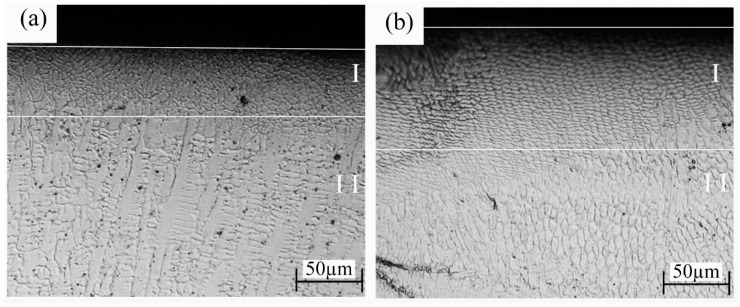
The metallographic images of the cross-sections of coating samples, (**a**) UB(FeCoNiCr), (**b**) UB(CoCrNi).

Figure 8 displays the metallographic structures of the longitudinal section of the UB-treated coating samples. It can be seen that the equiaxed-grain structure at the top of the coating disappears, and the fine-grained microstructure forming in the UB-treated coating samples takes its place. The surface influence layer of FeCoNiCr and CoCrNi MEAs is divided into two zones; namely, the grain refinement zone of Zone I and the unaffected zone of Zone II. Zone I refers to the outermost surface layer, in which the FeCoNiCr and CoCrNi MEAs form severe plastic deformation (SPD) layers with a thickness of 41.25 μm and 92.5 μm, respectively, and the grains are obviously refined. In Zone II, there is no obvious change in grain size owing to the greater distance from the processing surface.

In the process of UB, the rolling head impacts the coating surface under the co-action of ultrasonic vibration and static pressure, leading to the severe plastic deformation of the coating surface, accompanied by plastic deformations such as crystal plane slip, twinning, and grain boundary migration, the formation of high-density dislocation tangles and dislocation walls, as well as the emergence of grain in a streamlined structure [28]. As a result, the processing quality of the alloy surface is improved with residual compressive stress, and the refined gradient structures are formed in the surface layer. Furthermore, the FeCoNiCr and CoCrNi MEAs have a single FCC structure with more slip systems than BCC and HCP, which is easier to slide to form a streamlined structure in the deformation process [35,36].

### 3.4. Micro-Hardness and Elastic Modulus

**Figure 9:**
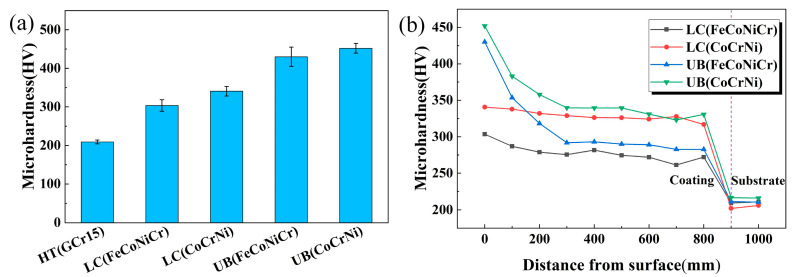
Micro-hardness results. (**a**) Surface hardness; (**b**) cross-section hardness variations.

The surface micro-hardness values of the substrate sample and four kinds of coating samples are displayed in Figure 9a. From the test results, the surface micro-hardness values of the LC(FeCoNiCr) and LC(CoCrNi) coatings were 303.5 HV and 340.6 HV, increasing by 45.2% and 62.9%, respectively, in comparison with the substrate. Furthermore, the micro-hardness values of the UB(FeCoNiCr) and UB(CoCrNi) MEA coatings reached 430HV and 452HV, increasing by 41.7% and 32.7%, respectively, compared to their corresponding untreated coatings. Hence, within the experimental scope, UB treatment could significantly improve coating hardness.

Figure 9b presents the cross-section micro-hardness variations of four coatings. It is found that the hardness distributions of the two untreated coatings were relatively even from the top surface to the coating–substrate interface, implying that the microstructures of the two untreated coatings were even. In comparison, the micro-hardness distributions of two UB-treated coatings presented a gradient change from the top surface down to the coating–substrate interface. Meanwhile, in all cases, the highest micro-hardness was distributed at the top surfaces. UB treatment is a kind of severe plastic deformation (SPD) technique. During UB, the top surface materials contact the burnishing roller and are subjected to the most severe deformation, thus having the finest grains, as evident in Figure 8. It is easy to surmise that the material plastic deformation resulting from dislocation by UB was decreasingly severe from the top surface down to the coating–substrate interface. Therefore, as seen in Figure 8, for the two kinds of UB-treated coatings, fine equiaxed grains formed on the top surfaces, and the grain sizes increased in an in-depth direction beneath the top surfaces. According to the Hall–Petch relationship [37,38], finer grains mean higher hardness. Therefore, a gradient hardness distribution formed in the cross-sections of two UB-treated coatings.

**Figure 10:**
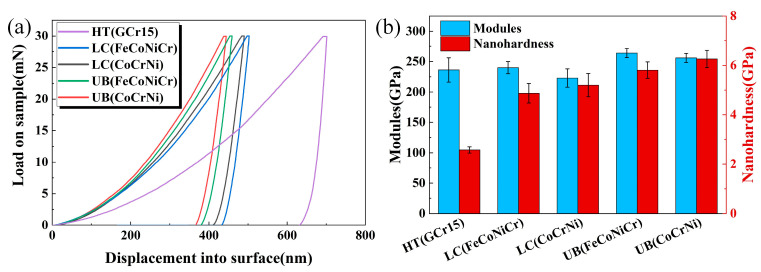
Nano-indentation test results. (**a**) Load–displacement curves; (**b**) elastic moduli and nano-hardness values.

In order to accurately characterize the mechanical properties of the four studied coatings, a nano-indentation test was carried out. As a result, the loading and unloading load-displacement curves are shown in Figure 10a. During the loading process, there were no obvious differences in the load–displacement curves of the five test coatings with a depth of penetration less than 50 nm, ascribing to the indentation size effect [39]. As the test began, the indenter made contact with partial surface asperities. With the indentation depth increasing, the asperities deformed plastically under the load. After the asperities were completely flattened by the indenter, the nano-indentation behavior of the coating was ultimately determined by its mechanical properties [40]. As shown in Figure 10a, under the maximum load of 30 mN, the maximum indentation depths of the HT(GCr15), LC(FeCoNiCr), LC(CoCrNi), UB(FeCoNiCr), and UB(CoCrNi) coatings were 701 nm, 503 nm, 490 nm, 459 nm, and 444 nm, respectively. Compared with the substrate material (HT(GCr15)), the plastic deformations of four coatings decreased. Meanwhile, the plastic deformations of the two UB-treated coatings were further reduced in comparison with the corresponding untreated coatings. Among them, the indentation depth of the UB(CoCrNi) coating was the minimum. As stated above, by UB treatment, the coating grains were refined, resulting from severe plastic deformation and thus high-density dislocations of materials. As known, finer grains meant more grain boundaries and therefore stronger resistance to deformation. Meanwhile, in the case of small grain size, under the external force, there would occur plastic deformation mostly inside grains, leading to uniform deformation and a small indentation depth.

The nano-hardness and elastic modulus obtained through nano-indentation are two important parameters to characterize coating properties. Generally, the bearing capacity and wear resistance of the coating can be reflected by the ratio of the hardness to elastic modulus, i.e., H/E and H^3^/E^2^, respectively, because they are stronger with the values of H/E and H^3^/E^2^ increasing [41]. Figure 10b shows the nano-hardness values of different samples. The surface nano-hardnesses of the HT(GCr15), LC(FeCoNiCr), LC(CoCrNi), UB(FeCoNiCr), and UB(CoCrNi) samples were 2.578 GPa, 4.871 GPa, 5.201 GPa, 5.808 GPa, and 6.259 GPa, respectively. For the two kinds of coating materials, the nano-hardness values of UB-treated coatings improved by about 20% in comparison with their corresponding untreated coatings, which was in good agreement with the results of the micro-Vickers hardness test in Figure 9a. The elastic moduli of different samples are presented in Figure 10b. Similar observations can be found for the two kinds of coating materials. That is, although there was a slight increase in the elastic modulus after UB treatment, such an increase was much less significant in comparison with that in nano-hardness. As can be seen from the test results, the grain refinement and residual stress induction caused by UB treatment played a significant role in the increase in coating hardness. However, the coating elastic modulus was not obviously changed.

### 3.5. Friction Wear

**Figure 11:**
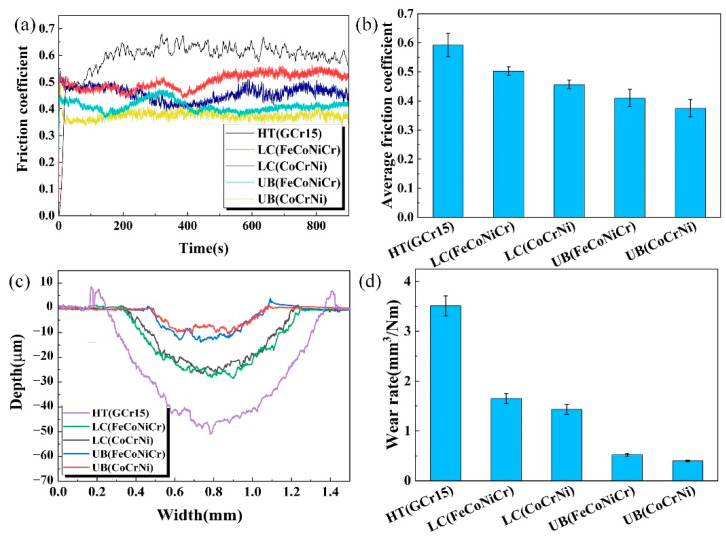
Friction test results. (**a**) Friction coefficient variations; (**b**) average friction coefficient; (**c**) wear profile curve of the cross-section; (**d**) wear rates of samples.

Figure 11a,b displays friction coefficient variations against time, as well as the average friction coefficients of the five different samples, respectively. As exemplified in Figure 11a, the friction coefficients of the five kinds of samples experienced a short running-in period at the very beginning of the wear test with rapid rising, then reached a steady state. It can be seen that when compared with the substrate sample, the friction coefficient fluctuations of four coatings decreased to varying degrees. In particular, the UB(CoCrNi) coating had the shortest run-in period and the minimum fluctuation. As shown in Figure 11b, the average friction coefficients of the HT(GCr15), LC(FeCoNiCr), LC(CoCrNi), UB(FeCoNiCr), and UB(CoCrNi) samples were 0.592, 0.503, 0.456, 0.410, and 0.375, respectively. Among them, the friction coefficient of the UB(CoCrNi) sample was the lowest. As stated above, there was grain refinement and surface work hardening after the UB treatment. Due to the surface smoothening and hardening effect resulting from UB, during wear, the contact area between the counterpart and coating surface became small, thus decreasing the friction coefficient [42,43].

Figure 11c,d shows the cross-section profiles of the wear scars and wear rates of the five tested samples. In Figure 11c, there formed bulges on both sides of the wear traces of the substrate sample and the two untreated coating samples, suggesting that extrusion and plastic flow occurred during wear. It is clear that the wear trace of the substrate sample had the highest bulges. Notably, the two UB-treated coating samples almost had no bulges, ascribing to the work-hardening effect of the UB treatment. As such, the metal flow was weaker after work hardening, with a difficulty in plastic deformation [8]. In terms of wear size, the substrate sample had the most serious wear, as well as the largest wear width and depth among all the samples. The wear width and depth of two untreated coating samples became smaller. Similarly, the wear widths and depths of the two UB-treated coating samples were further reduced in comparison to those without UB treatment, implying that the UB-treated samples had better wear resistance. The wear rates of all samples were calculated and are presented in Figure 11d. As can be seen, the wear rates of FeCoNiCr and CoCrNi untreated coatings were 1.65 mm^3^/Nm and 1.43 mm^3^/Nm, respectively, so their wear resistances were close to each other. Compared with the wear rate of the substrate sample (3.51 mm^3^/Nm), they decreased by 53.0% and 59.3%, respectively. Likewise, the wear rates of the UB-treated FeCoNiCr and CoCrNi coatings were 0.52 mm^3^/Nm and 0.40mm^3^/Nm, respectively, and decreased by 68.5% and 72.0% in comparison to those before UB treatment, illustrating that the UB treatment had a role in reducing the friction coefficients of FeCoNiCr and CoCrNi coatings. According to Archard’s law, wear rate is inversely proportional to hardness [44], so high hardness, as well as a large elastic modulus, were beneficial to improving the wear resistance of the FeCoNiCr and CoCrNi coatings.

**Figure 12:**
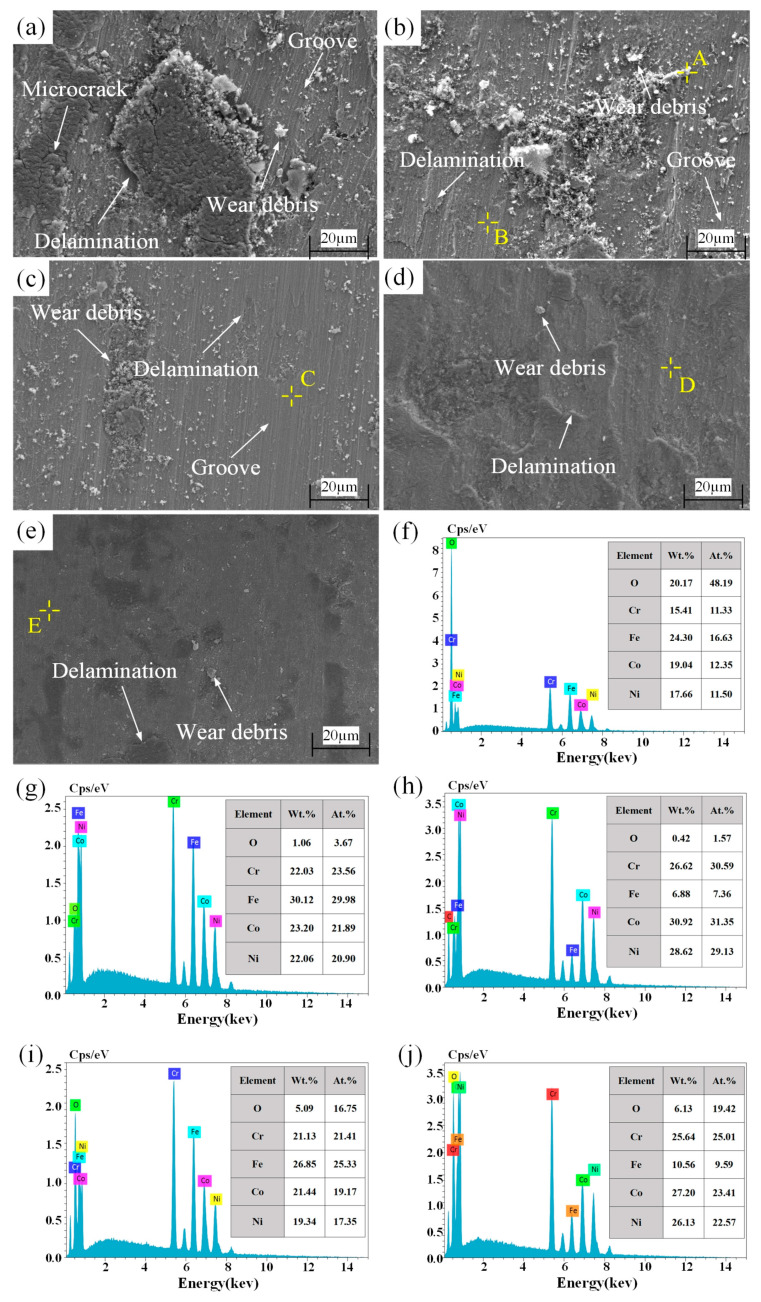
SEM images of wear areas and EDS analysis. (**a**) HT(GCr15), (**b**) LC(FeCoNiCr), (**c**) LC(CoCrNi), (**d**) UB(FeCoNiCr), (**e**) UB(CoCrNi), (**f**) element analysis of wear debris A of LC(FeCoNiCr), (**g**) element analysis of wear scar B of LC(FeCoNiCr), (**h**) element analysis of wear scar C of LC(CoCrNi), (**i**) element analysis of wear scar D of UB(FeCoNiCr), and (**j**) element analysis of wear scar E of UB(CoCrNi).

Figure 12a–e presents the SEM images of the wear areas of five kinds of samples. As shown in Figure 12a, the substrate sample showed obvious wear trace, with the main characteristics of micro-cracks, deep grooves, delamination, and wear debris. In the process of friction, micro-cracks easily occurred as the shear stress induced by friction exceeded the yield strength of the sample. The delamination mainly resulted from micro-crack propagation and coalescence. Furthermore, during the wear process, under the co-action of the normal load and shear force of the friction pair, the wear debris exfoliated from the sample surface, causing adhesive wear. The exfoliated wear debris embedded in the sample surface under the normal load. Later, the surface was scratched under shear load, thus accelerating the abrasive wear and then forming the parallel deep grooves. Hence, it is suggested from the wear characteristics that the wear mechanism of the substrate sample was mainly abrasive wear and adhesive wear.

As can be seen from Figure 12b,c, compared with the substrate sample, there was no visible crack found in the wear area of the LC(FeCoNiCr) and LC(CoCrNi) samples, and the delamination and grooves were obviously alleviated. Moreover, the size of the wear debris decreased. The reason for the wear resistance improvement of the untreated coating samples was the high surface hardness. A hard surface could hinder the deformation of the material, reducing micro-cracks as well as weakening the delamination wear. It can be found from Figure 12d,e that the wear of the two UB-treated coating samples was greatly weakened and the quantity and volume of wear debris in the wear areas decreased, and the grooves were shallowed compared with their corresponding untreated coating samples. The grains of the two UB-treated coating samples were refined via UB treatment, and their hardness and yield strengths were significantly improved. Thereby, the volume and quantity of wear debris produced in the wear test were reduced, and the abrasive wear was alleviated [45,46].

In order to determine whether there was oxidation wear in the wear process, the EDS analysis was carried out for the wear debris A and wear scars B, C, D, and E of the coating samples, as shown in Figure 12b–e. The element contents at A–E are illustrated in Figure 12f–j, and clearly, there was oxidation wear at all points A–E. During the oxidation wear, the oxidation film generated was ductile and softer than the substrate. Under the shear force between the friction pair, partial oxidation films would be exfoliated, and the exfoliated films could be smeared on the sample surface, serving as a solid lubricant and thereby reducing wear [47]. As shown in Figure 12f,g, the O element content of wear debris A was obviously higher than that at B without wear debris, which confirmed that the oxidation film was continuously exfoliated to form wear debris. In Figure 12g–j, the O element content at D and E was much higher than that at B and C, respectively, suggesting that the oxidation wear of the UB-treated coatings was more obvious. This is because the grain refinement, dislocation, and high internal stress generated from the UB treatment could make the oxidation film denser and thicker [48]. In addition, according to Archard’s law, the larger the load was in the wear process, the higher the hardness and the higher the temperature of the sample surface [49]. More importantly, oxidation wear easily occurs under high temperatures [50]. Hence, the UB treatment could enhance the oxidation wear, beneficial to improving the wear resistance of the coating surface.

## 4. Conclusions

This study adopted UB treatment for the surface strengthening of FeCoNiCr and CoCrNi coatings prepared by laser cladding. The results paved the way for the preparation and post-treatment of FeCoNiCr and CoCrNi coatings. By analysis, the conclusions were as follows:

(1) In comparison with the LC(FeCoNiCr) and LC(CoCrNi) coating samples, the surface roughness and porosity of the two corresponding UB-treated samples decreased significantly, illustrating that UB treatment could greatly smoothen the coating surface, decrease the porosity of coatings, and reduce surface defects. During UB treatment, under the applied external dynamic load, the materials in the coating surface and near-surface were forced to flow from surface peaks to valleys, and thereby the coating surface was flattened and the pores and defects in the coating surface were filled/cured.
(2) The Fe content in two kinds of coatings obviously increased due to the dilution effect of substrate on the MEAs during laser cladding. The microstructure of the two kinds of MEA coatings showed a plane crystal at the bottom, a columnar crystal (cellular dendritic crystal for CoCrNi coating) at the middle, and an equiaxed-grain crystal at the top. For the two kinds of coating, after UB treatment, there was obvious grain refinement within 100 μm beneath the top surfaces of the coatings, which resulted from dislocation accumulation from the severe plastic deformation of the materials.
(3) Compared with the LC(FeCoNiCr) and LC(CoCrNi) samples, the surface hardness and yield strength of the two corresponding UB-treated coating samples were improved. In both cases, after UB treatment, a gradient hardness structure was formed along the in-depth direction of the coating. Meanwhile, the two kinds of UB-treated coating samples exhibited better friction and wear properties than their corresponding untreated samples with wear rates decreasing by 68.5% and 72.0%, respectively.

## Figures and Tables

**Table 1:** Chemical compositions of the powders and substrate (At%).

Sample	C	Co	Cr	Mo	Ni	Si	Mn	P	S	Cu	Fe
FeCoNiCr powder	/	Bal.	22.58	/	25.66	/	/	/	/	/	24.60
CoCrNi powder	/	33.95	32.42	/	Bal.	/	/	/	/	/	/
Substrate	0.98	/	1.42	0.05	0.08	0.22	0.25	0.006	0.011	0.03	Bal.

**Table 2:** Laser cladding parameters.

Laser Power (W)	Laser Beam Diameter (mm)	Scanning Speed (mm/s)	Powder Feed Rate (mm/s)	Overlapping Ratio (%)
2000	4	1500	38.4	25

**Table 3:** UB parameters.

Frequency (kHz)	Feed Rate (mm/min)	Amplitude (mm)	Spindle Speed (rpm)	Pressure Deep (mm)
28	10	10	160	0.3

## Data Availability

The data presented in this study are available from the corresponding author upon reasonable request.
